# Case report: Genetic diagnoses in a pediatric patient with retinoblastoma and comorbid global developmental delay: three distinct entities diagnosed by whole exome sequencing in a single patient

**DOI:** 10.3389/fnins.2024.1391596

**Published:** 2024-07-23

**Authors:** Jing Chen, Shuo Yang, He Wang, Hongjing Wang, Yuanyuan Xiao, Shanling Liu

**Affiliations:** ^1^Department of Medical Genetics, West China Second University Hospital, Sichuan University, Chengdu, Sichuan, China; ^2^Key Laboratory of Birth Defects and Related Diseases of Women and Children, Sichuan University, Ministry of Education, Chengdu, Sichuan, China; ^3^Department of Obstetrics and Gynecology, West China Second University Hospital, Sichuan University, Chengdu, Sichuan, China

**Keywords:** whole exome sequencing, retinoblastoma, *RB1*, global developmental delay, case report

## Abstract

**Background:**

The objective of this study was to explore the genetic etiology and propose a genetic diagnosis and counseling strategy for children with retinoblastoma (RB) and global developmental delay (GDD).

**Case presentation:**

We report on a 2 years and 4 months old boy with binocular retinoblastoma and global developmental delay (included intellectual disability, language development delay, motor development delay, etc.). Genomic DNA was extracted from peripheral blood mononuclear cells isolated from the proband and his parents. Whole exome sequencing (WES) was carried out for the proband and his parents to identify genetic etiology, which was subsequently verified by quantitative polymerase chain reaction (qPCR).The WES revealed a gross heterozygous deletion in the RB transcriptional corepressor 1 (*RB1*, OMIM:614041) gene, including exon 7–8, in the affected proband but not in his parents. Additionally, two pathogenic copy number variations (CNVs) were identified: a duplication at 7q11.23 and a microdeletion at 16p11.2-p12.2, respectively. Furthermore, the genomic qPCR analysis demonstrated a 50% reduction in the copy numbers of exon 7 and exon 8 in the *RB1* gene of the proband, as compared to those detected in his parents. Simultaneous variants in the *RB1* gene and two pathogenic CNVs can precisely explain the genetic etiology of the proband.

**Conclusion:**

The present study firstly reports a novel gross deletion variant of the *RB1* gene coexisting with two pathogenic CNVs in a pediatric patient with retinoblastoma and comorbid global developmental delay in China. Additionally, our findings strongly support the use of WES in pediatric patients with RB comorbid GDD, and WES is recommended as the first-tier test.

## Background

The most prevalent intraocular tumor in children is RB, which typically occurs before the age of five and results from the loss of both *RB1* alleles within the tumor. The estimated incidence of RB is between 1 in 15,000 and 1 in 20,000 live births (Broaddus et al., 2009). This tumor exhibits uniqueness among central nervous system tumors, as it can be visualized through the eye without necessitating invasive imaging techniques. The most common initial manifestations of RB include leukocoria, a white reflex observed through the pupil, and strabismus. Recognizing these early indicators of RB is crucial for achieving favorable outcomes in affected children. However, in regions with low awareness of these signs and limited access to healthcare, diagnosis of RB may be delayed, often presenting with proptosis. RB can affect one or both eyes and occasionally involve the pineal, parasellar, or suprasellar regions as well (Dimaras and Corson, 2019). The dysfunction of the *RB1* gene is the predominant genetic etiology underlying RB. *RB1* gene, the first cloned tumor suppressor gene on chromosome 13, acts as a negative regulator of the cell cycle by binding to the transcription factor E2F and inhibiting the transcription of genes required for S phase (Hanahan and Weinberg, 2000). To date, 1,247 variants in *RB1* gene for variant class “Damage Mutation” have been documented in the Human Gene Mutation Database.^1^ However, the patients with *RB1* variant currently do not exhibit any clinical manifestations of global developmental delay (GDD).

Global developmental delay, as characterized by the inability to attain developmental milestones within the anticipated age-appropriate range. Objectively, this refers to a significant delay in two or more developmental domains in children aged 5 years or younger. Developmental domains encompass gross or fine motor skills, speech and language, cognition, personal-social, and activities of daily living. Intellectual disability (ID) involves impairments in general mental abilities that impact both intellectual functioning (such as learning and reasoning) and adaptive functioning (activities of daily living, including communication and independent living) (Bowling et al., 2017; Vasudevan and Suri, 2017).

The genetic etiology of GDD encompasses chromosomal disorders, microdeletion and microduplication syndromes, monogenic diseases, and mitochondrial disorders. Chromosomal microarray (CMA) commonly serves as the primary diagnostic genetic test for individuals with GDD, as copy number variations (CNVs) are the most prevalent cause. Whole exome sequencing (WES), however, is increasingly employed as a secondary genetic testing method.

In our study, WES revealed two CNVs, a duplication at 7q11.23 and a microdeletion at 16p11.2-p12.2, respectively. The duplication of 7q11.23 results in Williams-Beuren syndrome (WBS) (MIM:194050), with most duplications being *de novo* (Parrott et al., 2015). WBS is a unique neurodevelopmental disorder that can be identified based on several main clinical features, such as facial deformities, developmental delay (DD), ID, and supravalvular aortic stenosis (SAS), among others (Collins et al., 2010; Pober, 2010; Parrott et al., 2015). The prevalence of 7q11.23 duplication syndrome has been estimated to be between 1 in 7,500 and 1 in 20,000, with complete penetrance in both males and females (Van der Aa et al., 2009; Velleman and Mervis, 2011). However, patients with the 16p11.2-p12.2 microdeletion syndrome exhibit variable clinical manifestations, precluding the establishment of a distinct clinical feature. In addition, the penetrance for the microdeletion of 16p12.2 is incomplete (Girirajan et al., 2015). The diversity of clinical features published in patients with 16p11.2-p12.2 microdeletion syndrome includes cognitive impairment (ranging from mild to severe), DD, growth disorders, behavioral problems, as well as reports of asymptomatic cases (Zufferey et al., 2012).

Developmental delay and ID are common clinical manifestations of many genetic diseases, characterized by highly genetic heterogeneity. However, the coexistence of RB and GDD in a same patient is uncommon. The aim of this study is to investigate the genetic etiology of the proband with RB and complicated with GDD, and propose a diagnostic and counseling strategy.

## Case presentation

### Clinical data

**FIGURE 1:**
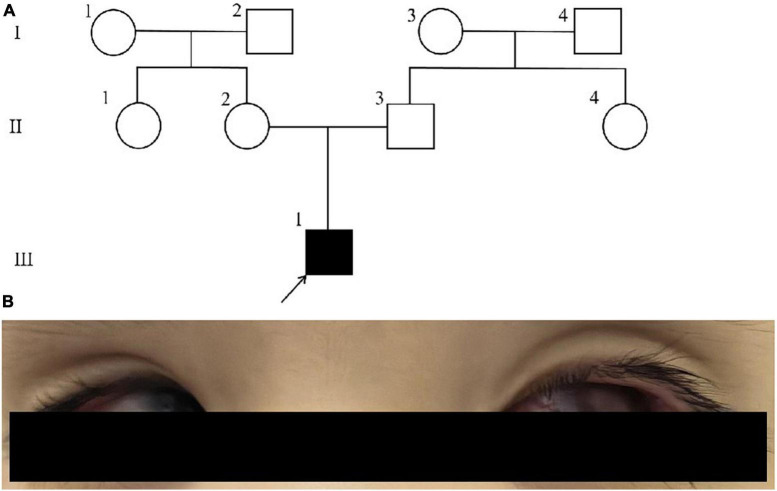
**(A)** The pedigree of this family. **(B)** The distinctive binocular phenotype exhibited by the proband.

The proband’s mother stated that there were no notable abnormalities during the prenatal examination. At 39 weeks and 1 day of gestation, a cesarean section was performed due to the fetus being in a breech position. At the age of 2+ months, no eye tracking was observed in both eyes, and an MRI scan revealed abnormal signal nodules in both eyeballs, indicating a diagnosis of binocular retinoblastoma. The proband is currently 2 years and 4 months old and remains visually impaired in both eyes and bilateral leukocoria, with a developmental delay. Refer to Table 1 for detailed information. The proband did not undergo any surgical procedures or receive other specific treatments, thus precluding the possibility of further genetic testing at the somatic cell level. The pedigree of this family and the binocular phenotype of the proband are depicted in Figures 1A, B. The written consent for publication of the article has been obtained from the proband’s parents. Informed consent has also been acquired from the parents regarding the inclusion of data and clinical details in this publication. The present study was granted ethical approval by the Medical Ethics Committee of West China Second University Hospital, Sichuan University (Chengdu, China).

**TABLE 1:** Patient characteristic and clinical features.

Patient	Proband
Variant	*RB1* exon 7-8 del; 16p11.2-p12.2 microdeletion syndrome; 7q11.23 duplication syndrome
Inheritance	*De novo*
Current age	2 years and 4 months
Age of diagnosis	2+ months
Gender	Male
Weeks of gestation	39^+1^ weeks
Facial deformity	Retinoblastoma
Ocular MRI	Abnormal signal nodules in the binocular:bulb:retinoblastoma in both eyes, no obvious abnormality in the binocular optic nerve, abnormal signal in the center of the bilateral semioval center:myelination insufficiency.
**Neurologic phenotype**	
Intellectual disability	Yes
Language development	No speech
Motor development	Unable to stand and sit alone
Behavior disorders	No
CT brain (at 3 months)	The bilateral lateral ventricles were dilated slightly, the extracerebral space in the fronto-parietal-temporal region was wider slightly, and the boundary between intracranial gray and white matter was unclear.
**Miscellaneous**	
Weight, height, and head circumference	Normal
Hearing loss	No
Feeding difficulties in infancy	No
Other system anomaly	No

### Exome sequencing and bioinformatic analysis pipeline

The total genomic DNA was extracted from peripheral vein blood of the proband and his family, utilizing the DNeasy Blood and Tissue Kit (Qiagen, Hilden, Germany). The exons and splice regions were captured and enriched using a Nano WES Human Exome V1 kit (Berry Genomics). A NovaSeq 6000 sequencing system with 150 paired-end reads (Illumina, USA) was used to sequence the captured library (average sequencing depth >100×). The reads were mapped to the human reference genome (hg38) using BWA (v0.7.15). Variants calling was performed by Verita Trekker software (v1.2.0.2).

The sequencing data annotation and pathogenicity classification were subsequently completed. The Genome Analysis ToolKit (GATK) pipeline was used for SNP/InDel calls, and the eXome Hidden Markov Model (XHMM) pipeline is used for CNV calls. Variants were classified into five categories: pathogenic (P), likely pathogenic (LP), uncertain significance (VUS), likely benign (LB), and benign (B), according to the guidelines of the American College of Medical Genetics (ACMG) (Richards et al., 2015).

### Quantitative polymerase chain reaction for *RB1* gene variant validation

Triplicate quantitative polymerase chain reaction (qPCR) was performed using genomic DNA to verify the WES results. SYBR Green qPCR Master Mix (Termo Fisher Scientific, Vilnius, Lithuania) and an Applied Biosystems 7500 Real-Time PCR System (Termo Fisher Scientific, Waltham, MA, USA) were utilized. The 2^–Δ(ΔCT)^ analysis method was used to evaluate the copy number of *RB1* exons 7 and 8 in each sample, employing the requested primer pairs (*RB1*-exon7-F:CAGTTAATGCTATGTGTCCTTG and *RB1*-exon7-R:ATCATCCTGTCAGCC TTAGA; *RB1*-exon8- F:AGTAGAAGAGGGATGGCAAA and *RB1*-exon8-R:GCACTC CTGT TCTGACCT).

## Results

**FIGURE 2:**
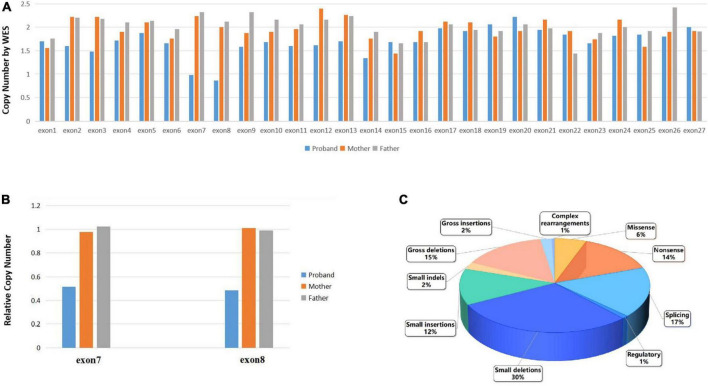
**(A)**. The CSCORE.CNV results following WES showed that the proband had only a single copy of exons 7 to 8 in the *RB1* gene, while the rest of the *RB1* gene had a copy number of two. Both maternal and paternal data indicated normal copy numbers for the entire *RB1* gene. **(B)** qPCR verification of the WES experiments revealed that the proband had half the number of copies for exon 7 and exon 8 compared to their parents. **(C)** The ratio of disease mutation types in the *RB1* gene which have been categorized as disease-causing mutations according to the Human Gene Mutation Database (HGMD^®^ Professional 2023.3).

Primarily, the single nucleotide variation (SNV) evaluation of the WES did not reveal any pathogenic or likely pathogenic variations associated with the subject’s disease phenotype. While the CNV evaluation of the WES revealed that the proband presented a heterozygous deletion of exon 7–8 in the *RB1* gene, with only one copy of these exons. The exon 1–6 and exon 9–27 were shown to have a copy number of two. Parental data showed that both the mother and father presented with two copies of each exon, as expected (Figure 2A). According to the guideline of ACMG (Velleman and Mervis, 2011), the heterozygous deletion of exon 7–8 of the *RB1* gene is a pathogenic variation associated with the phenotype of RB. Genomic qPCR analysis revealed that the proband exhibited a 50% reduction in copy numbers of exon 7 and exon 8 compared to those found in his parents (Figure 2B), with the raw data available in Supplementary Excel 1.

**FIGURE 3:**
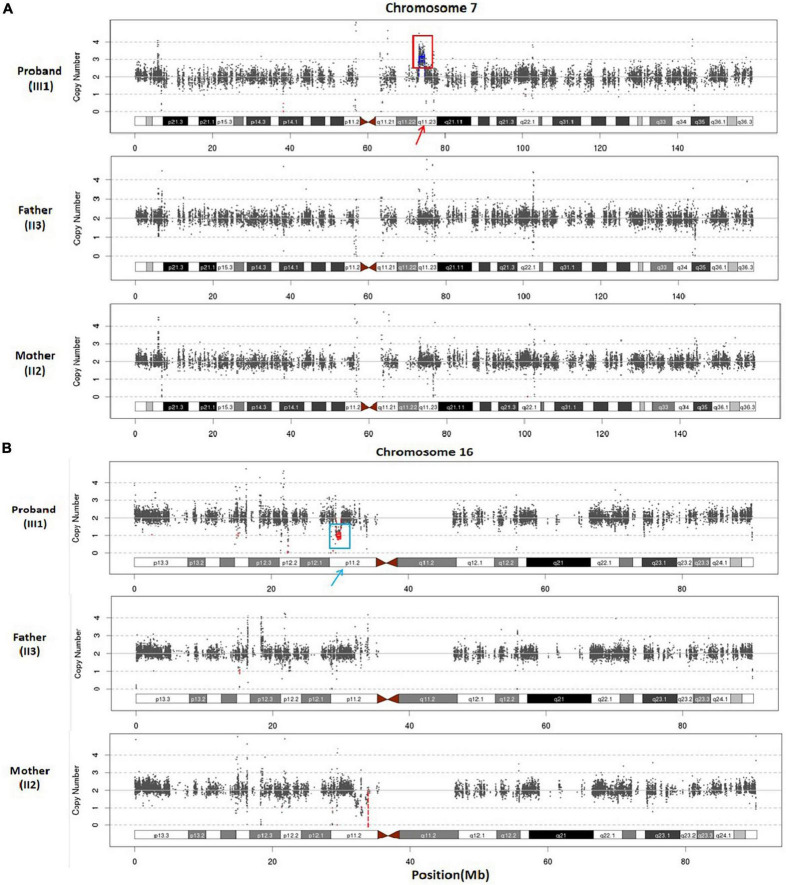
**(A)** Whole exome sequencing scatter plot was generated to illustrate the duplication of 7q11.23 (approximately 1.52 Mb) in the proband, and the parents exhibited normal copy numbers of this chromosome. **(B)** WES scatter plot was utilized to illustrate the heterozygous loss of 16p11.2, spanning approximately 0.65 Mb, in the proband, and the parents displayed normal copy numbers of chromosome 16.

Furthermore, WES analysis identified a 1.52 Mb duplication at chromosomal region 7q11.23 ([hg38]7q11.23 (73229566_74749542)x3) in the proband (Figure 3A). This duplicated segment encompasses a total of 39 genes, including 25 protein-coding genes. Notably, according to the ClinGen database, this duplicated chromosome segment completely overlaps with the 7q11.23 recurrent (Williams-Beuren syndrome) region (including ELN), indicating an established triplosensitive (TS) effect. Based on ACMG guidelines (Riggs et al., 2020), this microduplication at 7q11.23 scores one point and is classified as pathogenic CNV.

Additionally, WES analysis detected a heterozygous deletion spanning approximately 0.65 Mb within chromosome region 16p11.2 in the proband (Figure 3B), ([hg38]16p11.2 (29537167_30188698)x1). This deletion involves a total of 37 genes, including 27 protein-coding genes. Furthermore, the observed copy number deletion completely overlaps with an established haploinsufficient (HI) region known as the 16p11.2 recurrent region (proximal, BP4-BP5) (includes TBX6). According to ACMG (Riggs et al., 2020) criteria, the clinical significance of this 16p11.2 deletion is considered pathogenic. These two pathogenic CNVs provide an explanation for the genetic etiology underlying the developmental delay phenotype observed in the proband.

## Discussion and conclusion

Retinoblastoma is the most common intraocular malignant tumor in children, which affecting either the unilateral or bilateral eyes (Dimaras et al., 2015). The original cell of RB is most likely a developing cone photoreceptor precursor cell that undergoes loss of the *RB1* allele tumor suppressor gene and remains localized in the inner nuclear layer of the retina, potentially due to impaired migration toward the outer retina, thereby affecting its normal functionality (Rootman et al., 2013; Xu et al., 2014; Dimaras et al., 2015; Soliman et al., 2017). In approximately 50% of patients, the first copy of the *RB1* gene is compromised in most or all normal cells, leading to retinal tumors when the second copy of the *RB1* gene is also disrupted during retinal cell development (Dimaras et al., 2015). In the presence of a normal *RB1* gene, somatic amplification of MYCN oncogene can lead to RB in a small subset of patients. In various cell types, the loss of *RB1* gene can be compensated by an upregulated expression of related proteins (Rushlow et al., 2013). Nonetheless, the precursor of retinal cone cells, being a vulnerable cell, exhibits an inadequate compensatory mechanism, leading to unregulated cell proliferation and initiating carcinogenesis (Xu et al., 2014). In the Human Gene Mutation Database (HGMD^®^ Professional 2023.3), a total of 1,247 variants of the *RB1* gene in retinoblastoma have been documented and categorized as disease-causing mutations (DM). The majority of the variants consist of small deletions mutations (368/1,247, 30%), splicing (215/1,247, 17%), gross deletions mutations (188/1,247, 15%), nonsense mutations (174/1,247, 14%), and small insertions mutations (156/1,247, 12%) (Figure 2C). The deletion of exon 7–8 of the *RB1* gene identified in our study represents a gross deletion mutation.

The majority of patients carrying *RB1* gene variants exhibit nearly complete penetrance, while a minority demonstrate variable penetrance and expression, such as reduced tumor incidence and delayed onset (Soliman et al., 2016). The only way of early detection for RB is through a meticulous ocular examination conducted by a specialist, followed by corresponding genetic testing (Soliman et al., 2017). Early diagnosis of RB can preserve both a child’s life and visual function. However, anecdotal evidence indicates that numerous children worldwide are diagnosed at a late stage. The overall median age at diagnosis ranges from 11.2 to 36.5 months, with a median age of 23.5 months (Yousef, 2020). The diagnosis of RB in the proband occurred at 2+ months of age, which was earlier than the average age of disease detection. It is suggested that RB caused by exon 7–8 of the *RB1* gene exhibits an early onset age and bilateral involvement. However, it remains unclear whether the combination of pathogenic CNVs contributes to this particular scenario. Given that the first reported case with both conditions, further investigations into the underlying mechanisms are warranted in future studies.

Additionally, patients with germline variants in the *RB1* gene exhibit an elevated lifetime risk of developing secondary primary tumors, including osteosarcomas and soft tissue sarcomas. Therefore, it is imperative to enhance the clinical follow-up and consultation of these patients after establishing a definitive diagnosis of *RB1* germline variants through genetic testing (MacCarthy et al., 2013). And the proband in our study currently does not exhibit any extra-ocular tumors. Genetic testing for RB patients can facilitate the development of appropriate surveillance plans, thereby minimizing the need for expensive screening procedures among family members who are not at risk of inheriting pathogenic variants and providing crucial information for fertility counseling for affected family members (Soliman et al., 2017). The whole family experiences advantages via precise risk assessment based on genetic testing (Soliman et al., 2017).

The primary clinical manifestations of the 7q11.23 duplication syndrome encompass speech delay, motor delay, seizures, hypotonia, and DD, reinforced by ample evidence supporting the region’s TS. The duplication 7q11.23 syndrome is considered to be highly complete penetrant, and variable expressivity (Berg et al., 2007; Sanders et al., 2011; Velleman and Mervis, 2011). Patients with deletion of the proximal region at 16p11.2 (TBX6) also exhibit the following clinical features: DD, cognitive impairment, language delay, autism spectrum disorder, delayed language development, and obesity, among others (Zufferey et al., 2012; Hanson et al., 2014; Steinman et al., 2016). The patients with recurrent microdeletion of 16p11.2 have incomplete penetrance, and may be inherited from parents with subtle manifestations or those who remain asymptomatic. The penetrance of this deletion region was approximately 46.8%, as reported by Bijlsma et al. (2009) and Rosenfeld et al. (2013). Children with these two pathogenic CNVs and their families face many challenges in the realms of speech, language, and social behavior. Through comprehensive support, and targeted specialized interventions, the potential for a favorable prognosis among patients with these syndromes is enhanced (Velleman and Mervis, 2011).

In conclusion, we present a rare case of RB coexisting with GDD. This is the first reported case of a 2-year-old and 4-month-old male patient with a novel heterozygous deletion of exons 7–8 of the *RB1* gene, as well as two pathogenic CNV syndromes: 7q11.23 duplication syndrome and 6p11.2-p12.2 microdeletion syndrome, respectively. The concurrent presence of *RB1* gene variant and two CNVs can elucidate the etiopathogenesis underlying both RB and GDD in this child. Therefore, clinicians should recommend the most appropriate genetic testing method for counseling based on the specific family situation. Particularly in cases where the proband’s mother is currently pregnant, selecting an optimal testing method can allow for more time to conduct subsequent prenatal diagnosis. Furthermore, our study found no synergistic exacerbation between these two pathogenic CNVs in the proband nor any profound developmental delay observed. The potential cause could be attributed to the incomplete penetrance of these CNVs and variable severity. These findings provide valuable information for genetic diagnosis and counseling in patients with combined RB and GDD conditions. For patients presenting with both RB and GDD, WES should be considered as a first-tier test. The utilization of WES is not only essential for the rare combination of RB and GDD, but also for individual cases involving RB, GDD, and other related disorders. What is more, if the results of WES are negative, it is recommended to proceed with further whole genome sequencing (WGS).

## Data availability statement

The variation data reported in this paper have been deposited in the Genome Variation Map (GVM) (https://ngdc.cncb.ac.cn/gvm/) in National Genomics Data Center, Beijing Institute of Genomics, Chinese Academy of Sciences and China National Center for Bioinformation, under accession number GVM000807.

## Ethics statement

The studies involving humans were approved by the Medical Ethics Committee of West China Second University Hospital, Sichuan University (Chengdu, China). The studies were conducted in accordance with the local legislation and institutional requirements. Written informed consent for participation in this study was provided by the participants’ legal guardians/next of kin. Written informed consent was obtained from the individual(s), and minor(s)’legal guardian/next of kin, for the publication of any potentially identifiable images or data included in this article.

## Author contributions

JC: Writing – original draft. SY: Writing – original draft. HW: Writing – review & editing. HJW: Writing – review & editing. YX: Writing – review & editing. SL: Writing – review & editing.

## Footnotes

1
http://www.hgmd.org
